# Analysis of the synergistic benefits of typical technologies for pollution reduction and carbon reduction in the iron and steel industry in the Beijing–Tianjin–Hebei region

**DOI:** 10.1038/s41598-024-63338-8

**Published:** 2024-05-30

**Authors:** Wei Wen, Zifan Deng, Xin Ma, Yi Xing, Chongchao Pan, Yusong Liu, Han Zhang, W. A. N. D. Tharaka, Tongxin Hua, Liyao Shen

**Affiliations:** 1 School of Energy and Environmental Engineering, University of Science and Technology Beijing, Beijing, 100083 China; 2 CMA Earth System Modeling and Prediction Centre (CEMC), Beijing, 100081 China

**Keywords:** Iron and steel industry, Synergistic benefits, Emission inventory, WRF–chem, Environmental impact, Climate and Earth system modelling, Engineering

## Abstract

With its high energy consumption and pollutant emissions, the iron and steel industry is a significant source of air pollution and carbon emissions in the Beijing–Tianjin–Hebei (BTH) region. To improve air quality and reduce greenhouse gas emissions, a series of policies involving ultra-low emission, synergistic reduction of pollution, and carbon application have been implemented in the region. This study has assessed air pollutant and CO_2_ emission patterns in the iron and steel industry of the region by employing co-control effects coordinate system, marginal abatement cost curve, and numerical modeling, along with the synergistic benefits of typical technologies. The results have demonstrated that: (1) the intensive production activities pertinent to iron and steel enterprises has contributed greatly to the emission in Tangshan and Handan, where the sintering process is the main source of SO_2_, NOx, PM_2.5_, and CO, accounting for 64.86%, 55.15%, 29.98%, and 46.43% of the total emissions, respectively. (2) Among the typical pollution control and reduction measures, industrial restructuring and adjustment of the energy-resource structure have led to the greatest effects on emission reduction. Technologies exhibiting great potential in emission reduction and high-cost efficiency such as Blast Furnace Top Gas Recovery Turbine Unit (TRT) need to be promoted. (3) In Tangshan city with the highest level of steel production, the iron and steel production activities contributed to the concentration of 30.51% of PM_2.5_, 50.67% of SO_2_, and 42.54% of NO_2_ during the non-heating period. During the heating period, pollutants pertinent to the combustion of fossil energy for heating have increased, while iron and steel induced emissions have decreased to 23.7%, 34.32%, and 29.13%, respectively. By 2030, it is speculated that the contribution of the iron and steel industry to air quality will be significantly decreased as result of successful implementation of ultra-low emission policies and typical synergistic reduction technologies.

## Introduction

The iron and steel industry plays a vital role in the development of the national economy, providing a solid foundation for the high-quality growth of China’s economy^1^. Currently, China’s crude steel production has undergone a phase of rapid growth, securing its place as the world’s top steel producers. Notably, crude steel production in the Beijing–Tianjin–Hebei (BTH) region accounts for over 25% of the national output. Due to the complexity of its production process and high energy consumption, it also serves as a significant source of pollutants in the BTH region^2^. Pollutants emitted from the iron and steel industry into the atmosphere will further diffuse and transmit, leading to chemical reactions in the atmosphere to cause adverse effects on local air quality^3,4^. On the other hand, owing to intricate optical properties of pollutants, the industry can directly scatter or absorb solar radiation, disturbing the energy balance through aerosol-radiation interactions^5^. In the short term, aerosols emitted by the iron and steel industry alters meteorological factors such as downward radiation flux at the surface, near-surface temperature, wind speed, boundary layer height, etc. Accumulation of atmosphere pollutants over a long period can lead to changes in regional climate factors^6–8^. Research on the synergistic benefits of air pollution and climate has garnered significant attention from scholars^9^. The concept of “synergies” has also been introduced as early as the IPCC’s third report^10^, which refers to the reduction of localized pollutant emissions in conjunction with the reduction of greenhouse gas (GHG) emissions, or the GHG abatement benefits resulting from the reduction of pollutants^11^. Therefore, analyzing the synergistic benefit of typical technologies in the iron and steel industry in the BTH region and identifying the corresponding emission reduction pathways can offer crucial support for pollution prevention and control.

The 14th Five-Year Plan for Ecological Environmental Protection has highlighted the importance of promoting synergies between pollution reduction and carbon emission reduction, which serves as the key to realizing a comprehensive green transformation of the economy and society. Policies such as the “Implementation Plan for the Reduction of Pollutants and Carbon Synergy” has explicitly called for actions to decrease air pollutants, promote energy conservations, and reduce carbon emissions in the iron and steel industries. Conducting a synergistic assessment of typical technologies in the iron and steel industry is crucial for achieving the goal of reducing pollution and carbon emissions^12,13^. Various methodologies for evaluating synergistic effects have also been studied. Mao et al.^14^ has proposed three evaluation methods for co-control effects: the coordinate system, co-control cross-elasticity analysis, and unit pollutant abatement cost, all of which have been also applied to the iron and steel industry and other sectors for synergistic control assessment. Gao et al.^15^ has proposed the synergy effect assessment index method to determine the synergy effects. Wang^16^ has evaluated the synergistic effect of greenhouse gas and local air pollutant emission reduction in Tianjin by constructing a synergistic effect evaluation model. Deng^17^ has developed a partial equilibrium model to assess the synergistic emission reduction effect of CO_2_, SO_2_, and NOx. Further analysis of the emission reduction potential and cost assessment of synergistic control technologies can assist in selecting the optimal approach. Yang et al.^18^ has evaluated 28 energy-saving technologies in the iron and steel industry in the Yangtze River Delta region using energy-saving supply curves and scenario analysis, establishing a comprehensive bottom-up dynamic optimization model to simulate 48 development pathways for energy-saving and emission reduction technology. The iron and steel industry is a significant source of industrial pollution in the BTH region and the country as a whole. Although methods for assessing synergistic effects have been widely employed, there is still a lack of research on evaluating the synergistic control effect for emission reduction technologies of air pollution and CO_2_ in the iron and steel industry, especially in the BTH region.

Identifying emission characteristics, establishing an inventory of pollution sources, and analyzing emission contributions along with numerical models are essential for synergistic control of pollution reduction and carbon reduction. China has initiated the compilation of air pollution emission inventories in the 1980s, with related studies also conducted in the iron and steel field^19^. There are three methods for establishing emission inventories: the online monitoring method, pollution source investigation method, and emission factor method^20^. Zhou et al.^21^ has compiled a multi-sectoral pollution emission inventory, including the iron and steel industry in Jiangsu Province, relying on the emission factor method. With the use of continuous online monitoring data of pollutants and environmental statistics, Bo et al.^22^ has established a nationwide pollution emission inventory based on the main production processes in the iron and steel industry. Tang et al.^23^ has developed an emission inventory of the iron and steel industry in China of year 2018 by using a bottom-up method, and assessed the impacts on atmospheric condition. Li et al.^24^ has employed a combination of the emission factor method and GIS technology to establish an emission inventory of air pollution sources in the iron and steel industry in Tangshan. When integrated with numerical models, emission inventories can simulate the generation and dissipation of air pollution, offering technical support for air pollution prevention and control^18^. Duan et al.^2^ has utilized a bottom-up approach to establish a detailed multi-pollutant emission inventory of the iron and steel industry in the BTH region for the year 2015, using WRF-CAMx model to simulate the emission impact on the regional PM_2.5_ concentration. A detailed emission inventory contributes to an accurate understanding of the characteristics of pollution emissions from the iron and steel industry, forming the basis for the development of emission reduction policies^25–27^. However, there is still a lack of effort to develop a multi-pollutant emission inventory at process level in the BTH region under the new policy of synergistically reducing pollution and carbon.

To evaluate the synergistic control effect for pollution and carbon emission, along with its impact on air quality under the new policy, this paper is aimed to develop a pollutant emission inventory of the iron and steel industry in the BTH region using the emission factor method to analyze emission characteristics. The study has selected 10 typical technologies from the optimization of industrial structure, adjustment of energy structure, and energy-saving and emission reduction for assessing the synergistic effect. On this basis, the study has constructed scenarios for pollution and carbon reduction. Simultaneously, numerical models have been applied to analyze the impact of iron and steel pollutant emissions on air quality under these scenarios. The relevant findings can provide theoretical support for the future promotion of energy-saving technologies in the iron and steel industry in the BTH region. The study results and relevant technology also provide basis for future scientific research on source apportionment of air pollution and the synergistic emission reduction effect of climate change.

## Materials and methods

### Establishment of emission inventories

This study utilized investigation and research to obtain the pollution production factors, average control efficiency, and activity levels of steel enterprises in the BTH region in 2015. Determining the emission factors required production factors and control efficiencies. The production factors were mainly obtained from Technical Manual for the Preparation of Urban Air Pollutant Emission Inventories and related literature^28,29^. The emission control efficiency of pollutants were determined based on information in Steelmaking Industry Factor Manual in the Manual of Methods and Factors for Accounting for Industrial Source Production and Emissions. In result, emission factors were collected and shown in Table 1. We calculated the 2015 pollutant emissions in the BTH region through top-down emission factor methodology. The formula was shown in Eqs. (1) and (2).

1 $$E_{e,p,i} = A_{e,p} \times EF_{e,i} \times 10^{ - 3}$$

2 $${\text{EF}}_{e,i} = PF_{e,i} \times \left( {1 - PC_{e,i} } \right)$$

where $E_{p,t,i}$ represented emissions of pollutant $i$ from production process $p$ in enterprise $e$; $A_{i,j}$ represented activity level of production process $p$ in enterprise $e$, which was obtained through research; $EF_{e,i}$ represented emission factors for pollutant $i$ in enterprise $e$; $PF_{e,i}$ and $PC_{e,i}$ represented the production factors of the process, and the efficiency of pollution control, respectively.

**Table 1:** Pollutant emission factors for the iron and steel industry (g·Kg^−1^).

Process		Equipment scope	Organized^a^						Unorganized^b^
			SO_2_	NOx	PM_2.5_	VOC	CO	CO_2_	PM_2.5_
Coking		–	1.40	1.70	0.47	2.96	1.60	4588.24	0.18
Sintering		≥ 180m^2^	0.67	0.55	0.28	0.25	16.00	3137.95	0.09
		< 180m^2^	0.74	0.55	0.45				
Pelletizing		–	0.66	0.50	0.30	0.25	16.00	2462.82	0.09
Iron making		≥ 2000m^2^	0.07	0.17	0.63	0.00	15.29	2903.77	0.22
		< 2000m^2^	0.08	0.17	0.95				
Steel making	BOF	≥ 150t	0.00	0.00	0.31	0.05	8.75	–	0.32
		50t–150t	0.00	0.00	0.84				
	EAF	≥ 50t	0.00	0.00	0.18	0.06	9.00	–	0.18
		< 50t	0.00	0.00	0.48				
Rolling		–	0.003	0.00	0.086	0.00	1.28	3615.92	0.05

^a^Represents the pollution gas discharged through the exhaust pipe.

^b^Represents the pollution gas discharged irregularly without exhaust pipe.

### Abatement costing

#### Emission reduction potential

The emission reductions for a typical technology were established by the emission reduction factor and the level of activity. The formula was shown in Eq. (3):

3 $$E_{j, i} = B \times Q_{j, i}$$

where ${\text{E}}_{j, i}$ represented emission reductions from technology $j$ for pollutant $i$. ${\text{B}}$ represented crude steel production. ${\text{Q}}_{{\text{j ,i }}}$ represented the emission reduction of per unit production for pollutant $i$ after the implementation of technology $j$, which factor referred to Zhao^30^.

Emission reduction potential referred to the future emission reductions that can be achieved with the development and diffusion of technologies $j$. The total emission reduction potential of energy-saving and emission reduction technologies was calculated as shown in Eq. (4):

4 $$RP_{j, i} = E_{j, i} \times r_{j}$$

where ${\text{RP}}_{j, i}$ represented the total abatement potential for pollutant $i$ after the implementation of technology $j$; $r_{j}$ represented technology promotion rate.

This study utilized the calculation of the Integrated Contaminant Emission Reduction potential (ICER) to evaluate comprehensive emission reduction effects. The values selected in this paper were shown in Table 2, which were updated based on the research of Gao et al.^31^. The ICER was calculated as:

5 $$\begin{aligned} RP_{j, ICER} & = W_{LAP} \times ER_{LAP} + W_{GHG} \times ER_{GHG} \\ & = W_{LAP} \times \mathop \sum \limits_{j = 1}^{J} \alpha_{j} \times ER_{j} + W_{GHG} \mathop \sum \limits_{j = 1}^{J} \beta_{j} \times ER_{j} \\ & = W_{LAP} \times \left( {\alpha_{1} ER_{{SO_{2} }} + \alpha_{2} ER_{{NO_{x} }} + \alpha_{3} ER_{{PM_{2.5} }} } \right) + W_{GHG} \times \beta_{1} \times ER_{{CO_{2} }} \\ \end{aligned}$$

where $ER_{LAP}$ represented air pollutant emission reductions; $ER_{GHG}$ represented greenhouse gas emission reductions; $ER_{j}$ represented the emission reduction of pollutant $j$; $\alpha_{j}$ represented the coefficient by which emission reduction of air pollutant $j$ was converted to $ER_{LAP}$; $\beta_{j}$ represented the coefficient that converted the greenhouse gas emission reduction to $ER_{GHG}$, for which only CO_2_ was considered in this study ($\beta_{1}$ = 1); $W_{LAP}$ and $W_{GHG}$ represented the coefficients of $ER_{LAP }$ and $ER_{GHG}$ converted to ICER, respectively.

**Table 2:** Selection of parameters for ICER calculations.

Parameter		Value	Clarification
$W_{LAP}$		6 yuan/LAPeq	According to the Environmental Protection Tax Law, the tax for air pollutant ranges from 1.2 to 12 yuan/LAPeq^a^. The study set 6 yuan/LAPeq
$W_{GHG}$		0.0046 IAPeq/kgCO_2_eq	This study used the average price of carbon emissions trading piloted in 8 provinces and cities from 2017 to 2020 for discounting^b^, which is 0.02735 yuan/kg. According to the pollutant tax and carbon price, we obtain the value of $W_{GHG}$.$W_{GHG} = \frac{0.02735}{6} = 0.0046\;{\text{IAPeq}}/{\text{kgCO}}_{2} {\text{eq}}$
SO_2_	$\alpha_{1}$	1/0.95 LAPeq/kg	The air pollutant equivalent conversion factor is the reciprocal of the pollution equivalent. According to the Environmental Protection Tax Law, pollution equivalent values for SO_2_, NOx, and PM_2.5_ were 0.95 kg, 0.95 kg, and 2.18 kg, respectively^c^
NOx	$\alpha_{2}$	1/0.95 LAPeq/kg	
PM_2.5_	$\alpha_{3}$	1/2.18 LAPeq/kg	
CO_2_		$\beta_{1} = 1$	Global Warming Potential (GWP) uses CO_2_eq as a conversion factor, so the value is taken as 1

^a^Data from appendix 1 of the Environmental Protection Tax Act.

^b^Data from the Carbon Trading Platform of China (http://www.tanjiaoyi.com/).

^c^Data from appendix 2 of the Environmental Protection Tax Act.

#### Cost of emission reduction technology

The Marginal Abatement Cost Curve (MACC) served as an important method for assessing climate change policies and determining optimal paths^32–34^. This method integrated the abatement potential and the abatement costs, and it was defined as the ratio of the incremental cost of the abatement technology to the abatement potential. The cost of abatement technologies included investment and operating costs, energy saving benefits, and abatement benefits. The formula^30^ was provided as following:

6 $$C_{j,i } = \frac{{C_{j} }}{{Q_{j ,i } }}$$

7 $$C_{j} = I_{A,j} + I_{AO,j} - E_{j} - AP_{j}$$

8 $$I_{A,j} = I_{j} \times \frac{{d \times \left( {1 + d} \right)^{n} }}{{\left( {\left( {1 + d} \right)^{n} - 1} \right)}}$$

9 $$E_{j} = S_{e,j} \times P_{e} + S_{pe,j} \times P_{pe}$$

10 $$AP_{j} = \partial \times RP_{{{\text{SO}}_{2} }} + \beta \times RP_{{{\text{NOx}}}} + \gamma \times RP_{{{\text{PM}}_{2.5} }} + \delta \times RP_{{{\text{CO}}_{2} }}$$

where $C_{j}$ represented the technology cost of applying technologies $j$; $I_{A,j}$ represented the investment cost of the technology $j$; $I_{AO,j}$ represented the annual operating investment cost of the technology; $E_{j}$ represented the energy efficiency of the technology $j$; $AP_{j}$ represented the emission reduction benefits of the technology $j$; $I_{j}$ represented the cost of construction; $n$ represented the repayment period, and relevant data were obtained from the results of the study by Ren^35^; $d$ represented the discount rate, taken as 20%; $S$ represented energy savings per unit of technology; $P$ represented energy prices per unit, where $e$ and $pe$ stand for electricity and primary energy, respectively, and primary energy prices, electricity prices were weighted average prices of coal and coke, and large industrial electricity prices ,which was 35 yuan/GJ and 0.84 yuan/kW h respectively; ${\text{AP}}_{{\text{j}}}$ represented emission reduction benefits of the technology $j$; $\partial ,\upbeta ,\upgamma ,\updelta$ were weighting factors for SO_2_, NOx, PM_2.5_ and CO_2_ respectively, which was 5730 yuan/t, 6390 yuan/t, 48,980 yuan/t^36^,and 42.85 yuan/t^18^.

In MACC, the X-axis represents the abatement potential of technology for pollutant $i$, and the Y-axis represents the unit pollutant abatement cost of the technology. In this paper, 1.5 yuan per kilogram was used as the high and low abatement cost cut-off point. 0 yuan per kilogram was used as the positive and negative abatement cost cut-off point^32^. The measures with negative abatement cost were characterized by benefits outweighing costs. This type of technology can achieve pollutant emission reductions while being less costly compared to the other conventional technologies, making them worth for extensive promotion. The measures with low abatement costs resulted no benefit, but the investment cost for this type of technology was low. With the support of government subsidies and the enterprises’ own investment, these technologies can progressively undergo upgrade^32^. Measures with high abatement cost required further investment in scientific research and more pilot scale trial after cost reduction is achieved^37^.

### Assessment of synergy and effectiveness of pollution and carbon reductions

**Figure 1:**
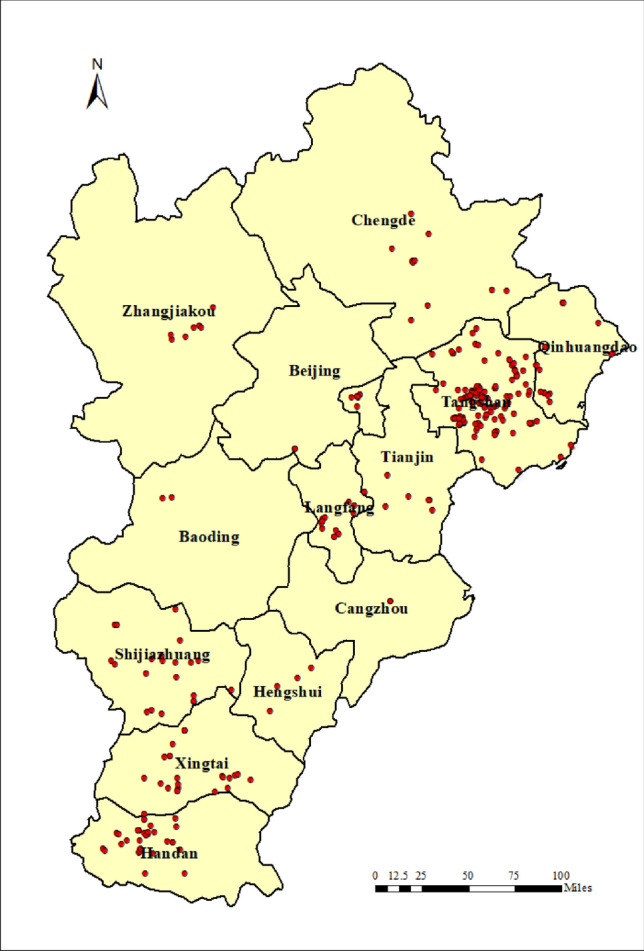
Spatial distribution of iron and steel enterprises in the BTH region. *Note*: This figure was generated by the authors by using Arc Geographic Information System (Arcgis) Vision10.8 (https://www.esri.com/zh-cn/arcgis/products/arcgis-desktop/overview).

In this study, Weather Research and Forecasting model coupled to Chemistry (WRF–chem) was used to assess the impact of pollutant emissions from the steel industry in BTH on air quality. The settings used in the modeling were referred to our previous studies^38^. This study applied meteorological data of year 2019 as the initial and boundary condition field, provided by the National Centers for Environmental Prediction (NCEP). July and December were selected for the simulation of non-heating and heating period, respectively. The anthropogenic emission input consisted of power, industry, residential, transportation, and agriculture, which obtained from emission inventory of year 2017 in MEIC (the Multi-resolution Emission Inventory for China) (http://meicmodel.org.cn). In this study, the grid emission data of the iron and steel industry in the BTH region were updated by the method introduced in “Establishment of emission inventories” section. Figure 1 showed the spatial distribution of iron and steel enterprises in the BTH region. On this basis, emissions have been updated for the 2030 scenario, taking into account of the successful implementation of ultra-low emission policy and the widespread adoption of representative low carbon technologies in 2030.

Table 3 showed the specific program settings.

**Table 3:** Simulation scenario setting.

Scenario	Description
Scenario 1	Deduction of iron and steel sources from industrial sources in the original MEIC inventory
Scenario 2	Deduction of iron and steel sources from industrial sources in the original MEIC inventory and addition of the iron and steel sector emissions inventory for 2015
Scenario 3	Deduction of iron and steel sources from industrial sources in the original MEIC inventory and addition of the iron and steel sector emissions inventory for 2030

We defined the impact of emission reduction on air quality as:

11 $$\Delta_{V1} = Scenario \,2 - Scenario \,1$$

12 $$\Delta_{V2} = Scenario \,3 - Scenario \,1$$

13 $$\Delta_{V*} = Scenario \,3 - Scenario \,2$$

where $\Delta_{V1}$ represented the impact of pollutant emissions from the iron and steel industry on air quality in BTH under the baseline scenario (scenario 2); $\Delta_{V2}$ represented the impact of pollutant emissions from the iron and steel industry on air quality in BTH under the pollution reduction and carbon reduction scenario (scenario 3); $\Delta_{V*}$ represented impact of implementation of ultra-low emission policy and adoption of typical technologies on air quality improvement compared with the baseline scenario.

## Results and discussion

### Pollution characterization of air pollutant and CO_2_ emissions

**Figure 2:**
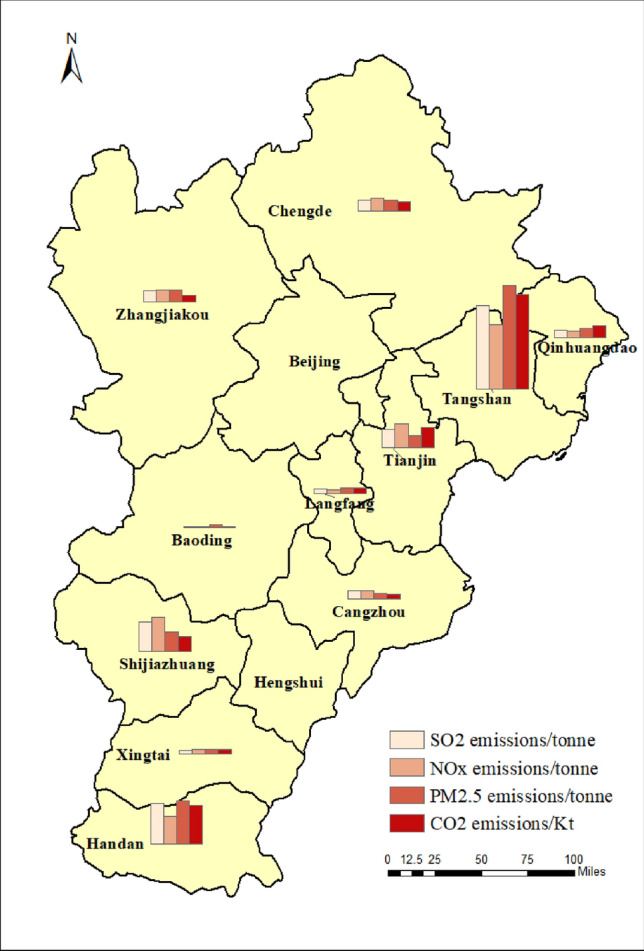
Distribution of enterprises and spatial distribution of major air pollutants pertinent to the iron and steel industry in BTH. *Note*: This figure was generated by the authors by using Arc Geographic Information System (Arcgis) Vision10.8 (https://www.esri.com/zh-cn/arcgis/products/arcgis-desktop/overview).

**Figure 3:**
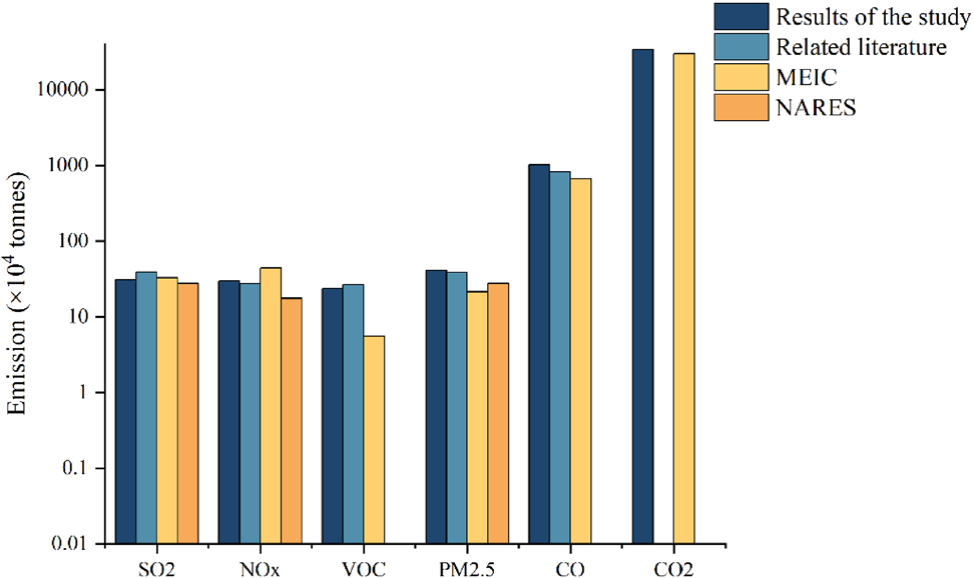
Comparison of the inventory results with existing studies.

The emission pertinent to the iron and steel industry in the BTH region were calculated by the method introduced in “Establishment of emission inventories” section. Figure 2 showed that the spatial distribution of major air pollutants from the iron and steel industry in BTH. 209.024 Mt of crude steel was produced in BTH, with the highest level of production in Hebei province, reaching 188.32 Mt. Among cities of Hebei, Tangshan was the largest crude steel producer, with 82.697 Mt accounting for 39.6% of the total production in BTH. The second largest producer, Handan, contributed 43.582 Mt, accounting for 20.9% of the total production. The total emissions of SO_2_, NOx, PM_2.5_, VOC, CO, and CO_2_ were 307.3 Kt, 296.0 Kt, 406.2 Kt, 235.4 Kt, 10,229.2 Kt, and 340,459.9 Kt, respectively. Due to the large number of iron and steel enterprises and their large scale production activities in Tangshan and Handan, the emission in those two cities were particularly high. The emissions in Tangshan accounts for 42.59%, 37.87%, 35.82% and 44.29% of total emission of PM_2.5_, SO_2_, NOx and CO_2_ in BTH, respectively, while emission in Handan made up of 17.74%, 15.25%, 15.65% and 18.01% of total emission of those parameters, respectively. However, the emission inventory established in this study have uncertainty, which was mainly ascribed to random errors from obtaining the activity levels data and selecting the emission factors, which may affect the accuracy of the inventory results. Therefore, it is necessary to compare the established emission inventory results with existing studies, including literature^2^, databased from MEIC and the National Annual Report of Ecological and Environmental Statistics (NARES) (https://www.mee.gov.cn/). According to the comparison shown in Fig. 3, similar distribution pattern of pollutant emissions was observed. The discrepancy could be mainly explained by difference in boundary condition and methods employed. For example, the calculation performed this study was primarily based on activity levels, while MEIC mainly utilized datasets from Continuous Emission Monitoring System (CEMS) and electric load. NARES obtained the emission level based on direct measurement in the enterprise operation, with more focus on ferroalloy related operations. In general, the inventory established in this study was generally consistent with the previously attained results, demonstrating great level of reliability of this inventory.

**Figure 4:**
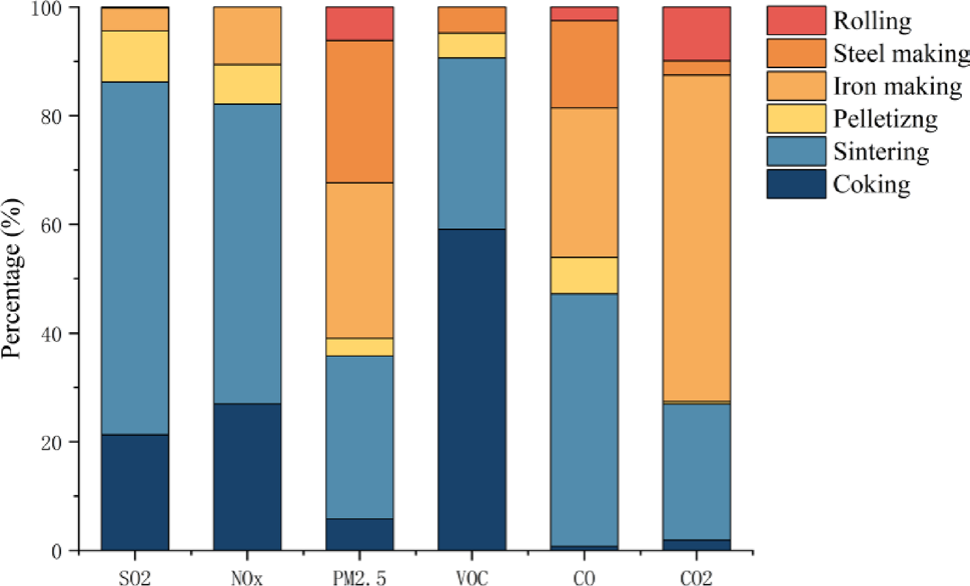
Proportion of pollutant emissions from different processes of iron and steel in BTH region.

Figure 4 showed the emission of pollutants from different production processes. SO_2_ and NOx were mainly emitted from sintering process, accounting for 64.86% and 55.15% of total emissions of these two parameters. Sintering, ironmaking and steelmaking processes accounted for 29.98%, 28.57% and 26.20% of total PM_2.5_ emission, respectively. VOC was mainly from sintering and coking, which made up of 31.52% and 59.11% of VOC emission, respectively. The relative contribution of sintering process to CO emission was 46.43%, while CO_2_ was mainly from ironmaking, accounting for 60.05% of total emissions.

**Figure 5:**
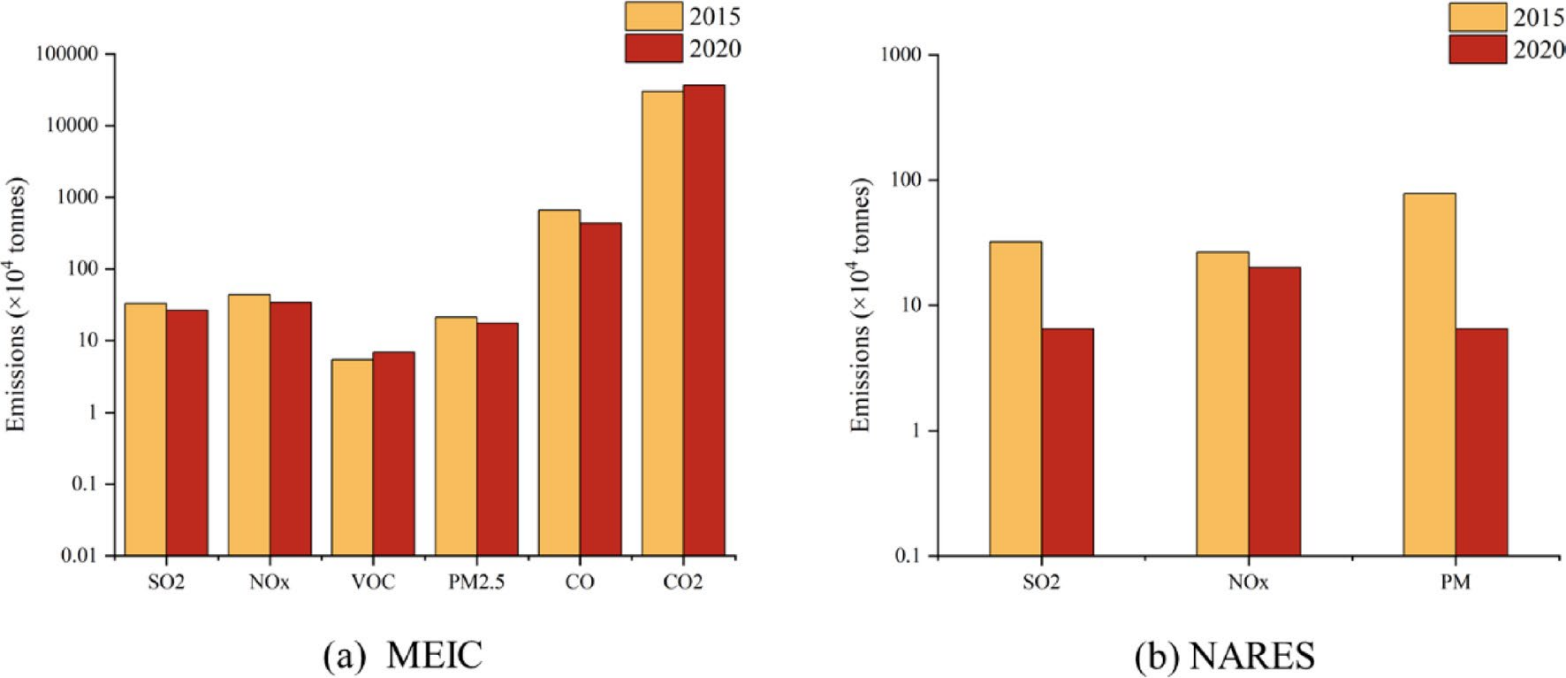
Comparison of pollutant emissions in 2015 and 2020.

On this basis, we calculated the pollution emissions from the iron and steel industry in the BTH region in 2020. Figure 5 showed the comparison of air pollutant emissions between 2015 and 2020. In 2020, new policies (e.g. “Opinions on Promoting the Implementation of Ultra Low Emissions in the Steel Industry” and “Iron and steel industry ultra-low emission transformation implementation plan”) was published to accelerate the implementation of ultra-low emissions in the steel industry^39,40^. To implement the new policy, iron and steel enterprises in the BTH region phased out the outdated production capacity, improved the performance efficiency of pollution treatment facilities, and achieved the peak production level. According to NARES, the emission of SO_2_, NOx and PM were 65.59 Kt, 200.34 Kt and 65.18 Kt in 2020, respectively. According to MEIC the emission of SO_2_, NO_x_, VOC, PM_2.5_, CO and CO_2_ were 65.4 Kt, 117.7 Kt, 115.9 Kt, 96.6 Kt, 9981.9 Kt and 350,079.4 Kt.Comparing with emissions in 2015, the results showed that the promotion of ultra-low emission retrofit enhanced the emission reduction of pollutants in the production chain. According to National Eco statistical Annual Report, SO_2_, NOx and PM were reduced by 80%, 25% and 92% from 2015 to 2020, respectively, as indicated in Fig. 5a. Figure 5b showed a results comparison based on MEIC inventories. According to MEIC, the reductions of SO_2_, NOx, PM_2.5_, and CO emission were 19.9%, 22.3%, 17.6%, and 34.6%, respectively. An increase in CO_2_ emission with a growth rate of 22.8% was observed. The robust level of crude steel production had led to a substantial amount of CO_2_ emissions. However, the emission control policy primarily targeted on other conventional pollutants during the 13th Five Year Plan period. According to China Statistical Yearbook 2021, crude steel production in 2020 was 271.49 Mt, which was 29.88% higher than level obtained in 2015. The iron and steel industry underwent enormous pressure to reduce CO_2_ emissions in the future. While addressing air pollutants, more attention needed to be given in BTH for synergistic CO_2_ emission reduction.

### Evaluation of typical technologies synergistic effects and cost-effectiveness

#### Selection of typical technologies and evaluation of synergistic effects

An integrated assessment of various policies (e.g. “Peak Carbon Implementation Program in Industry” and “Carbon Neutral Vision and Low Carbon Technology Roadmap for the Steel Sector”), “National Catalogue of Key Low Carbon Technologies for Promotion”, and related literature^24,30,33,41–43^ was performed to evaluate three major aspects of pollution and carbon reduction policies and technologies: industrial structure optimization, energy structure adjustment, and application of energy-saving technologies. The energy-saving technologies were studied with primary focus on coking, sintering, ironmaking, steelmaking, and rolling. The background information of the selected emission control measures were provided in Table 4.

**Table 4:** Description of the selected synergistic control measures.

Type	Number	Measures	Process	Background
Industrial restructuring	T1	Scrap—electric arc furnace steelmaking (Scrap-EAF)	–	This technology carried out short-flow processes and scrap recycling, leading to additional savings for sinter, pelletize, coke and ironmaking
Adjustment of energy ratios	T2	Hydrogen steelmaking	–	This measure substituted fossil fuel with hydrogen, reducing pollutant emissions
Energy saving and emission reduction technologies	T3	Coal moisture control (CMC)	Coking	By modifying the fluidized bed, this measure fully utilized the coke oven flue gas to moderately dry the raw coal
	T4	Coke dry quenching (CDQ)	Coking	This solution utilized recirculating gases to cool red-hot coke. The recovered sensible heat generated high-temperature, high-pressure steam was used
	T5	Flue gas waste heat recovery	Sintering, pelletizing	This technology utilized a high-efficiency, low-temperature waste heat boiler to convert waste flue gases produced by the equipment into steam to generate electricity
	T6	Dehumidification blast technology for blast furnace	Iron making	This technology utilized condensation for dehumidification. It removed saturated water by lowering the temperature of the humid air below the saturation temperature corresponding to the moisture content of the air
	T7	Blast Furnace Top Gas Recovery Turbine unit (TRT)	Iron making	This technology converted pressure and thermal energy into mechanical energy, which enabled the generator to produce electricity and realized energy recovery
	T8	Combined cycle power plant (CCPP)	–	This technology utilized a mixture of by-product gas and air for combustion. The produced gas with high temperature and high pressure drove the generator for power generation
	T9	Dry Recovery Technology for Converter Gas	Steel making	This technology involved cooling the high temperature flue gas and passing it through an electrostatic precipitator. The filtrated flue gas was recovered and the unfiltrated flue gas was ignited and discharged
	T10	Process control technology for hot rolling mills	Rolling	This technology combined big data analysis with mathematical modeling to adjust the hot rolling process while monitoring all key parameters of the process

**Figure 6:**
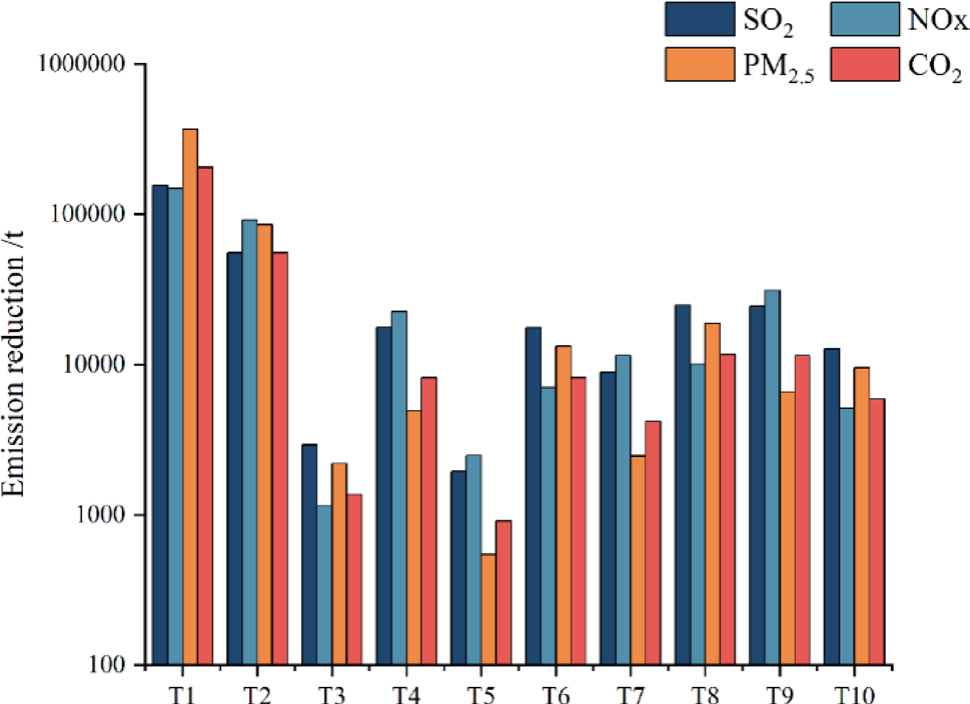
Emission reduction of air pollutants and CO_2_ by various pollution abatement measures.

Figure 6 displayed the emission reductions induced by different typical technologies, calculated by Eq. (3). Among the 10 listed emission abatement measures, industrial restructuring (i.e. T1) and adjustment of energy-resource structure (i.e. T2) exhibited the highest emission reductions level for all types of pollutants. Among the energy saving and emission reduction technologies, T8 and T9 showed the best emission reduction performance. T8 led to the largest PM_2.5_ emission reduction, accounting for 29.6% of the total technologically induced emission reduction. T9 resulted in the highest reduction of NOx, accounting for 33.2% of the total technologically induced reduction. Both T8 and T9 exhibited decent performance of emission reduction for SO_2_ and CO_2_, accounting for 21.1% and 20.8% of the total SO_2_ emission reduction, and 21.1% and 20.7% of the CO_2_ emission reduction, respectively.

This study utilized coordinate system analysis to evaluate the effectiveness of various technologies for synergistic control of air pollutants and greenhouse gases. This approach involved plotting the emission reductions performance of air pollutants (X-axis) and CO_2_ (Y-axis) achieved by the technology T1–T10 in a two-dimensional coordinate system. If a point fell within the first quadrant, it signified a synergistic reduction effect on both air pollutants and CO_2_. The farther away the points are from the origin, the better the synergy. An angle greater than 45 degrees between the line connecting the data points of the technology performance and the origin and the x-axis indicated that the technology resulted in greater reduction of air pollutants than CO_2_. Conversely, a smaller angle signified a stronger effect on CO_2_ reduction. Points in the third quadrant suggested that the technology increased emissions of both pollutants. Points in the second and fourth quadrant indicated a lack of synergistical effect^37^.

**Figure 7:**
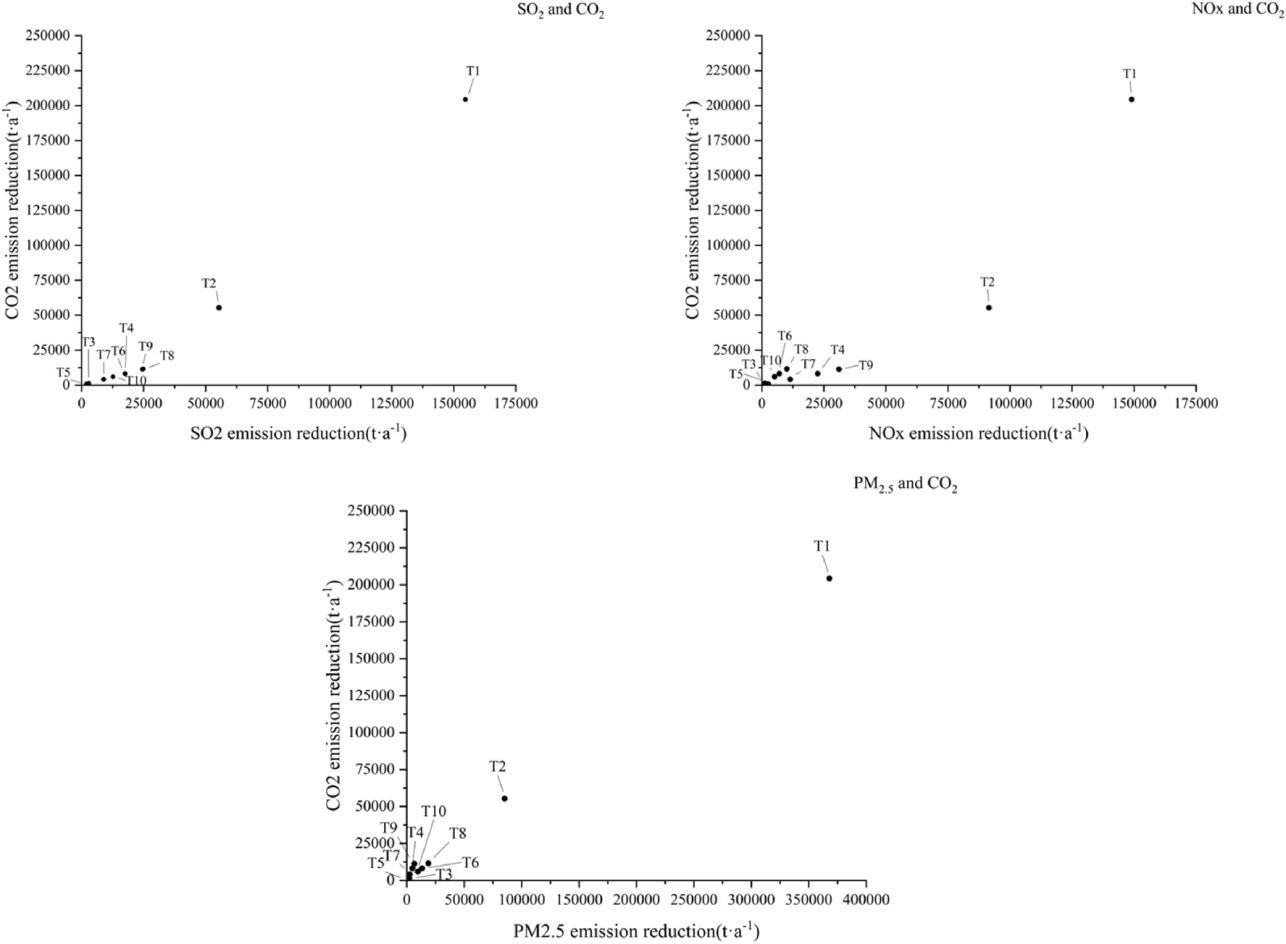
Coordinate system for synergistic control of air pollutants and CO_2_.

In Fig. 7, emission reductions performance induced by T1–10 were plotted into the coordinate system to display the degree of synergistic control effects. The results revealed that all measures are situated in the first quadrant of the coordinates system, indicating synergistic control effects of those typical abatement technologies on SO_2_, NOx, PM_2.5_ and CO_2_. Among all points, T1 and T2 were the farthest from the origin, indicating a pronounced synergistic effect on conventional pollutants (SO_2_, NOx, PM_2.5_) and CO_2_. Lines connecting T4, T7 and T9 to the origin formed less than 45 degrees angles with X-axis, implying greater efficiency of reduction for SO_2_ and NOx compared to CO_2_ emission reduction. In the coordinate system of synergistic control of PM_2.5_ and CO_2_, the angles formed by the line connecting T4, T7 and T9 to the origin and the X-axis were greater than 45 degrees. This contrasting trend suggested that the emission reduction of PM_2.5_ was relatively lower than that of emission reduction of CO_2_. Therefore, emission reduction of SO_2_ and NO_x_ were also greater than that of PM_2.5_ when removing the same amount of CO_2_.

#### Cost analysis of synergistic abatement of typical technologies

**Figure 8:**
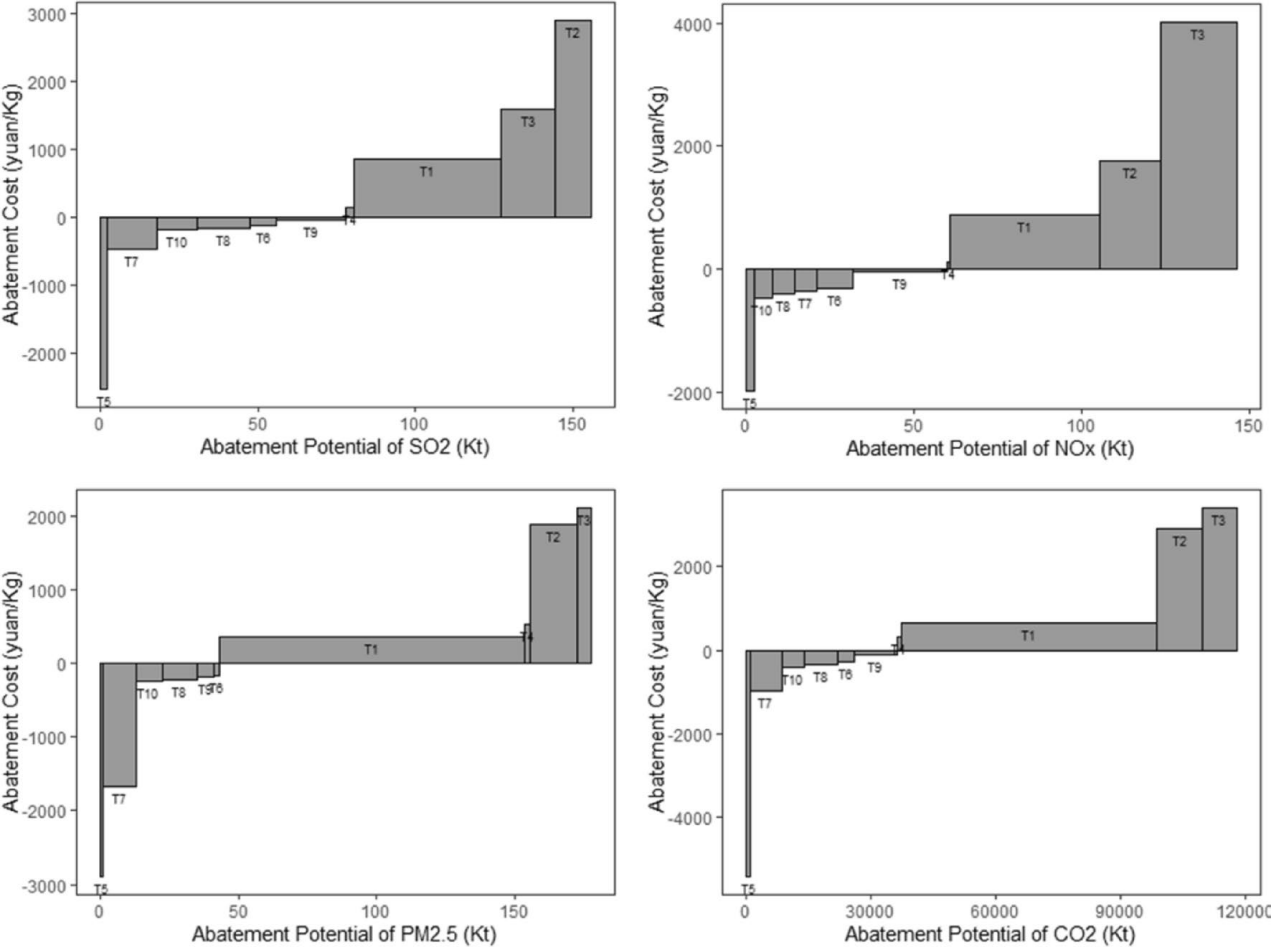
MACC of Air Pollutants and CO_2_ for iron and steel industry in BTH region.

The investment cost, cost of construction and energy savings per unit were determined by referring to relevant literatures^10,44–46^. Based on Eqs. (4)–(10), the abatement potential and abatement cost for each technology were calculated. Figure 8 provided the MACC of various abatement technologies. As introduced in “Cost of emission reduction technology” section, 1.5 yuan per kilogram was used as the high and low abatement cost cut-off point in this study. 0 yuan per kilogram was used as the positive and negative abatement cost cut-off point^32^. 6 technologies with negative abatement cost technologies (i.e. T5, T6, T7, T8, T9, T10) were identified. These technologies showed effective emission reduction performance for SO_2_, NOx, PM_2.5_, and CO_2_, they also achieved cost reduction. Among these technologies, T5 was the least costly technology for the reduction of SO_2_, NOx, PM_2.5_ and CO_2_, with marginal abatement costs of − 2523.16 yuan/Kg, − 1971.56 yuan/Kg, − 2885.86 yuan/Kg, and − 5394.14 yuan/Kg, respectively. However, the abatement potential of T5 was low. In the MACC for SO_2_, PM_2.5_ and CO_2_, T7 ranked with the second least cost only to T5, however, it exhibited greater abatement potential than T5, with emission reductions of 16.1 Kt, 12.2 Kt and 7556.8 Kt, respectively. In the MACC of NOx, the cost of implementing T6, T7, T8 and T10 were also relatively low, ranging from − 465.26 to − 303.53 yuan/kg. Among them, T7 and T8 exhibited the highest emission reduction potentials of 6.54 Kt and 6.78 Kt, respectively. Among the technologies with positive marginal cost, T1 and T2 demonstrated higher abatement potential for all types of pollutants, accounting for more than 36% of the total abatement.

**Figure 9:**
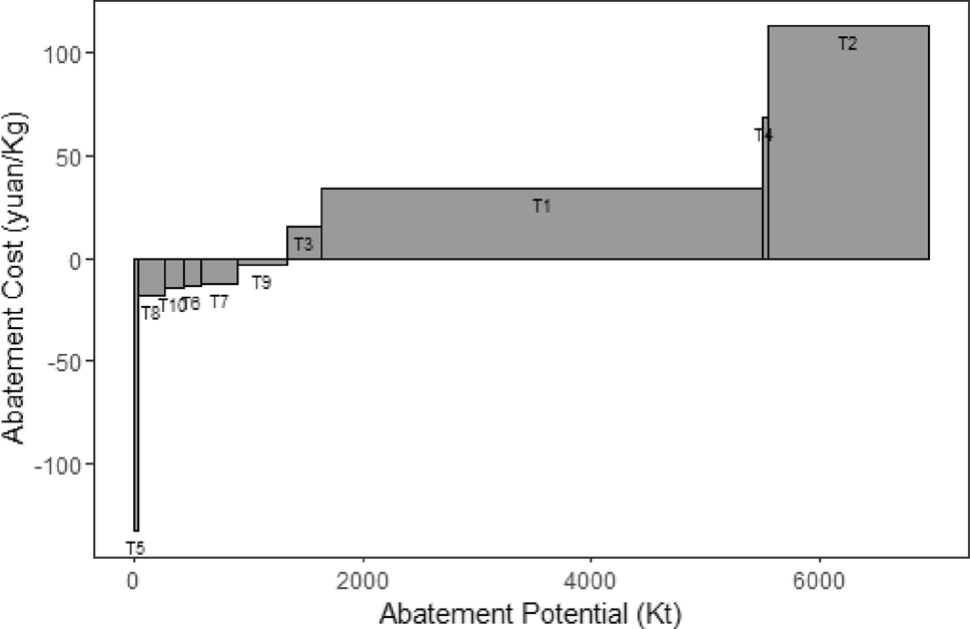
MACC of integrated pollutants for iron and steel sector in BTH Region.

In order to assess the cost-effectiveness of synergistic reductions in air pollution and greenhouse gases, Eq. (5) was used to calculate the reduction potential of integrated pollutants. The MACC of various measures for the combined pollutants were displayed in Fig. 9. According to calculation results, T5, T6, T7, T8, T9 and T10 were among abatement processes with negative abatement cost for dealing with integrated pollutants. T5 exhibited the lowest abatement cost of − 1971.56 yuan/Kg, while T7 and T9 showed the highest abatement potential, with reduction potential of 325.54 Kt and 421.00 Kt, respectively. In comparison, T1 and T2 incurred higher abatement cost, but it showed promising emission reduction result with 75.9% of pollutant reduction achieved. All six technologies with negative abatement cost involved comprehensive utilization of waste heat and waste energy, with the two abatement technologies exhibiting the highest abatement potential classfied under industrial structure and energy ratio adjustment. Therefore, in align with the demand for low-carbon and green development of the iron and steel industry, it would be crucial for BTH region to actively promote the comprehensive utilization of waste heat and energy technology. At the same time, continuously adjustment of industrial structure, optimization of the energy utilization, and the cost reduction were essential to achieve greater benefits.

### Analysis of *iron* and steel emission impact on air quality

In this section, WRF–chem model was used to assess the impact of iron and steel emission on air quality. As described in “Assessment of synergy and effectiveness of pollution and carbon reductions” section, three scenarios were established. Scenario 2 evaluated the impact of emissions from the iron and steel industry on air quality in the BTH region under base year (Eq. 11). Scenario 3 studied the effects of pollution and carbon synergy reduction (Eq. 12). In scenario 2, all ultra-low emission retrofits were completed and typical technologies were applied till 2030, which would have meet the requirements specified by “Opinions on Promoting the Implementation of Ultra-low Emission in the Iron and Steel Industry”. Typical technologies for synergistic reduction of pollution and carbon were evaluated mainly based on results of the cost curve in “Evaluation of typical technologies synergistic effects and cost effectiveness” section. The study selected three best synergistic technologies: Scrap-EAF, hydrogen steelmaking, and TRT. Hydrogen steelmaking was classified into hydrogen-based direct reduction and hydrogen-rich blast furnace smelting. Among them, hydrogen-rich blast furnace smelting was widely used^47^, and can be promoted as the main technology for steel emission reduction.

With the progressive development of technology, the effects of pollution and carbon reduction of each abatement technology had been gradually improved. Considering the data comparability and accessibility, the abatement factors reported by Zhao^30^ were utilized. Therefore, the effects of emission reductions were updated in this section. We also obtained the promotion of the technologies in the target year according relevant literatures^18,33^. The emission reductions for the resulted scenarios were calculated according to Eq. (4), with results presented in Table 5.

**Table 5:** Emission reductions from the iron and steel sector under the synergistic scenario of pollution reduction and carbon reduction/10^4^ t.

	Details	Rate (%)	SO_2_	NOx	PM_2.5_
Ultra-low emission retrofit	Based on the annual reports of the key iron and steel enterprises, we obtained the pollutant removal efficiency and updated the pollutant emission factors	100	24.19	17.83	30.96
Scrap-EAF	Pollutant emission intensity of SO_2_, NOx and PM_2.5_ was 0.05, 0.12 and 0.05 kg/t, respectively^48^	30	1.66	2.74	2.55
Hydrogen-rich blast furnace smelting	When the blowing volume of ammonia reached 100 m^3^/t, the coke usage can be reduced by 16.6 kg^49^	20	2.60	3.12	2.94
TRT	TRT generated electricity at 45 kW h/t^47,50^	100	1.23	1.11	1.20

**Figure 10:**
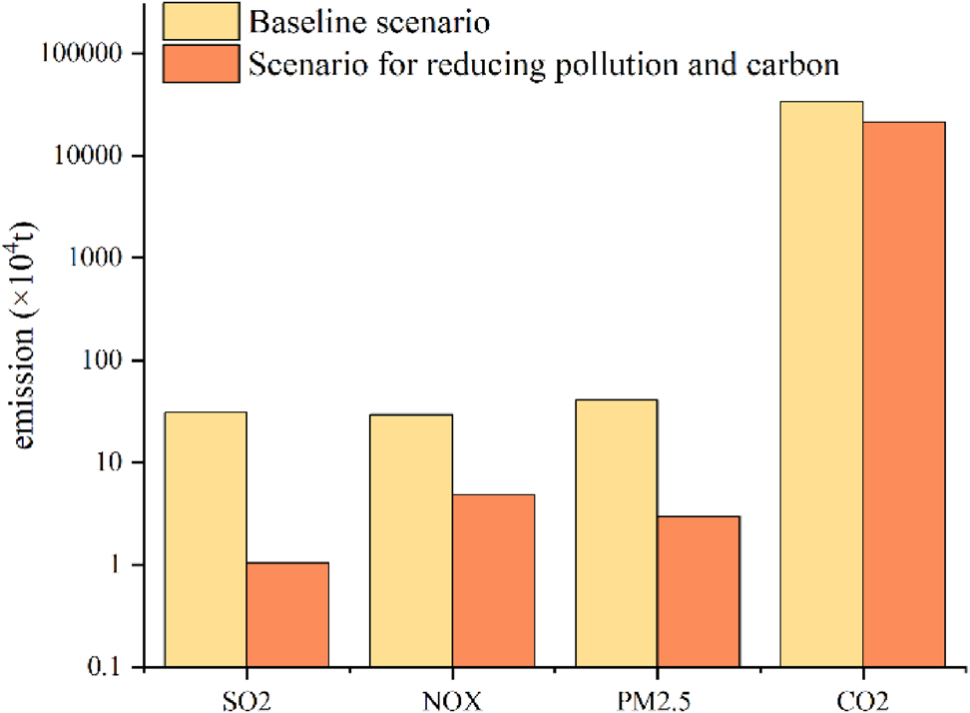
Comparison of pollutants emissions in BTH’s iron and steel industry between 2015 and 2030.

Emissions from iron and steel for the baseline scenario were described in “Pollution characterization of air pollutant and CO_2_ emissions” section. Figure 10 showed the comparison of pollutant emissions between the baseline and the synergy reduction scenario pertinent to pollution and carbon emission. To accomplish the synergistical effects of pollution and carbon reduction, enterprises completed the ultra-low emission retrofit and implemented the above three emission reduction technologies. The results showed promising emission reduction effects on SO_2_, NOx and PM_2.5_. At the same time, pollution and carbon reduction measures exhibited a significant effect on CO_2_ reduction. Compared with the baseline scenario, emissions of SO_2_, NOx, PM_2.5_ and CO_2_ from the iron and steel industry in BTH were reduced by 0.2968Mt, 0.248Mt, 0.3765Mt and 128.61Mt, corresponding to a reduction efficiencies of 96.58%, 83.78%, 92.69% and 37.78% respectively.

**Figure 11:**
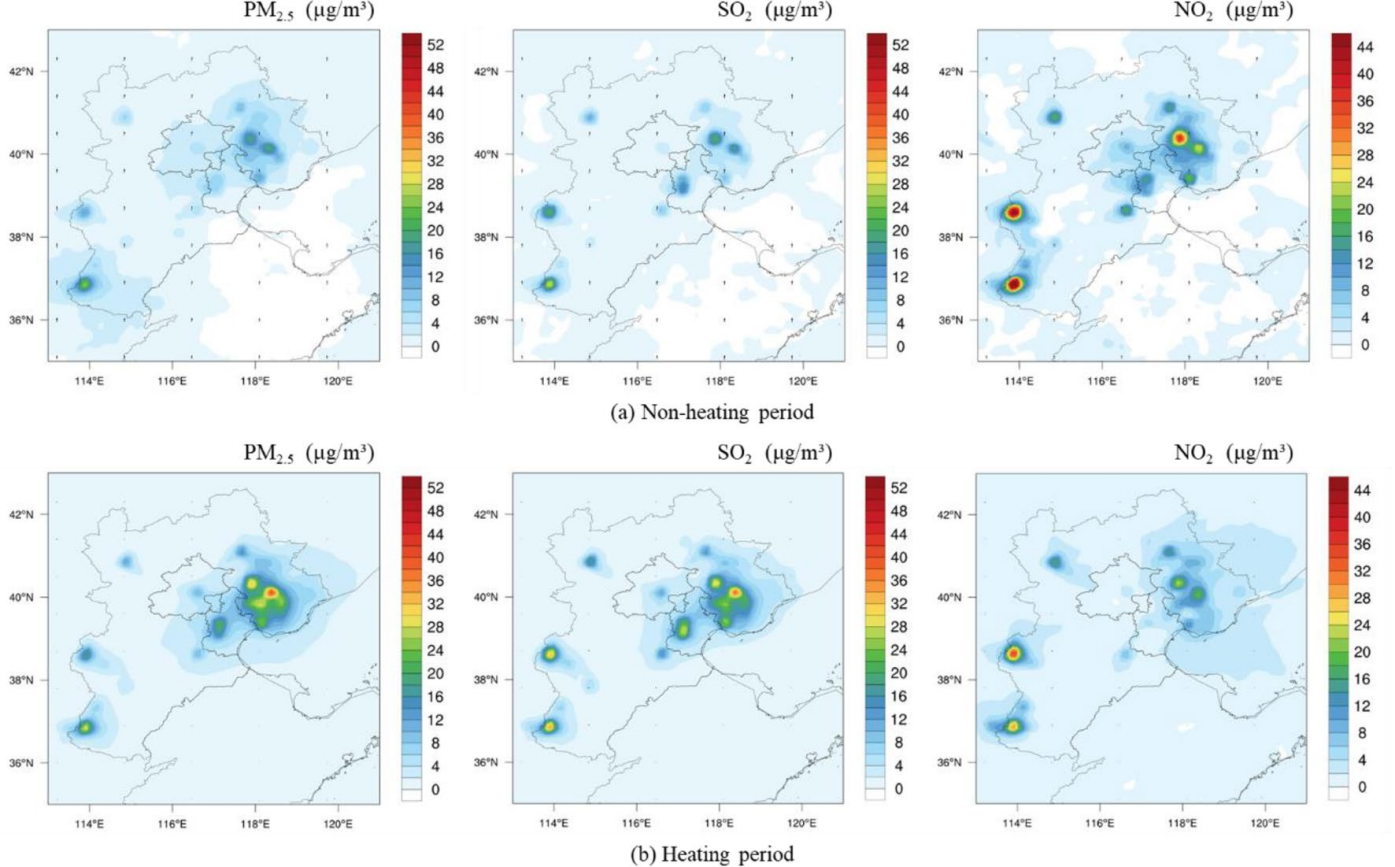
Air quality impacts of iron and steel industry emissions under baseline scenarios in the BTH region. *Note*: This figure was generated by the authors by using NCAR Command Language (Vision 6.4) (URL: 10.5065/D6WD3XH5).

On the basis of inventory calculation, the WRF–chem model was used to simulate the emission impact on air quality. Figure 11 illustrated the spatial distribution of the impacts on regional air quality (PM_2.5_, SO_2_, NO_2_) under base year (Eq. 11). Figure 11a showed the impact on air quality during non-heating period, and Fig. 11b showed the impact during heating period. Under the baseline scenario, the contribution of pollutant emissions from the iron and steel industry to PM_2.5_, SO_2_, and NO_2_ in the BTH region ranges from 0–24 μg/m^3^, 0–28 μg/m^3^, and 0–44 μg/m^3^ in the non-heating period. During the heating period, the contribution ranged from 0–46 μg/m^3^, 0–42 μg/m^3^, and 0–42 μg/m^3^, respectively. The major emission contribution mainly occurred around the cities with intensive industrial activities related to iron and steel enterprises, such as Tangshan, Shijiazhuang and Handan. This simulation was consistent with the spatial distribution of Fig. 2. The high iron and steel emission contributions were also due to inadequacy in implementation of ultra-low emission retrofit project in 2015, which resulted in a relatively low pollution control efficiency. The city such as Beijing had been less affected by iron and steel emission thanks to fewer iron and steel production operation in the city. The monthly average concentrations of PM_2.5_, SO_2_ and NO_2_ were 2.53 μg/m^3^, 2.34 μg/m^3^ and 3.36 μg/m^3^, respectively. However, the rise in PM_2.5_ in the neighboring areas due to elevated level of iron and steel industry operation would lead to obvious impact on Beijing’s air quality. Among cities with iron and steel production, the relative emission contribution to total PM_2.5_, SO_2_, and NO_2_ of BTH region were 30.51%, 50.67%, and 42.54% during non-heating period in Tangshan, respectively. In Shijiazhuang, the pollution output of iron and steel industry accounted for 30.74%, 43.43%, 47.47% of the total regional pollution concentration of PM_2.5_, SO_2_, and NO_2_, respectively. During heating period, the relative contribution of pollution emissions to total PM_2.5_, SO_2_ and NO_2_ were 25.31%, 37.03% and 41.24% in Shijiazhuang, respectively, while 23.7%, 34.32% and 29.13% were attributed to iron and steel production in Tangshan. The pollutants emitted from the combustion of fossil energy for heating increased due to the heating season demand. The production activities of iron and steel industries were restricted, which resulted in reduced emission. Table 6 showed the concentration contribution to air quality from iron and steel enterprises in key cities.

**Table 6:** Impacts on air quality in representative cities in the modeled region under the baseline scenario, the pollution reduction and carbon reduction scenario, and pollution abatement.

	Pollutant factor	Non-heating period			Heating period		
		$\Delta_{V1}$	$\Delta_{V2}$	$\Delta_{V*}$	$\Delta_{V1}$	$\Delta_{V2}$	$\Delta_{V*}$
Beijing	PM_2.5_ (μg/m^3^)	2.53	0.45	− 2.08	10.43	0.50	− 9.93
	SO_2_ (μg/m^3^)	2.34	0.41	− 1.93	10.71	0.27	− 10.44
	NO_2_ (μg/m^3^)	3.36	0.89	− 2.47	14.05	0.57	− 13.48
Shijiazhuang	PM_2.5_ (μg/m^3^)	15.09	1.99	− 13.1	11.69	1.42	− 10.27
	SO_2_ (μg/m^3^)	21.60	1.02	− 20.58	15.33	0.29	− 15.04
	NO_2_ (μg/m^3^)	40.80	4.49	− 36.31	33.00	1.79	− 31.21
Tangshan	PM_2.5_ (μg/m^3^)	22.05	3.40	− 18.65	33.75	3.96	− 29.79
	SO_2_ (μg/m^3^)	19.78	2.16	− 17.62	21.98	0.98	− 21
	NO_2_ (μg/m^3^)	20.40	6.60	− 13.8	22.13	1.06	− 21.07

where $\Delta_{V1}$ represented the impact of pollutant emissions from the iron and steel industry on air quality in BTH under the baseline scenario (scenario 2); $\Delta_{V2}$ represented the impact of pollutant emissions from the iron and steel industry on air quality in BTH under the pollution reduction and carbon reduction scenario (scenario 3); $\Delta_{V*}$ represented the impact of completion of ultra-low emission policy and diffusion of typical technologies on air quality improvement compared with the baseline scenario.

**Figure 12:**
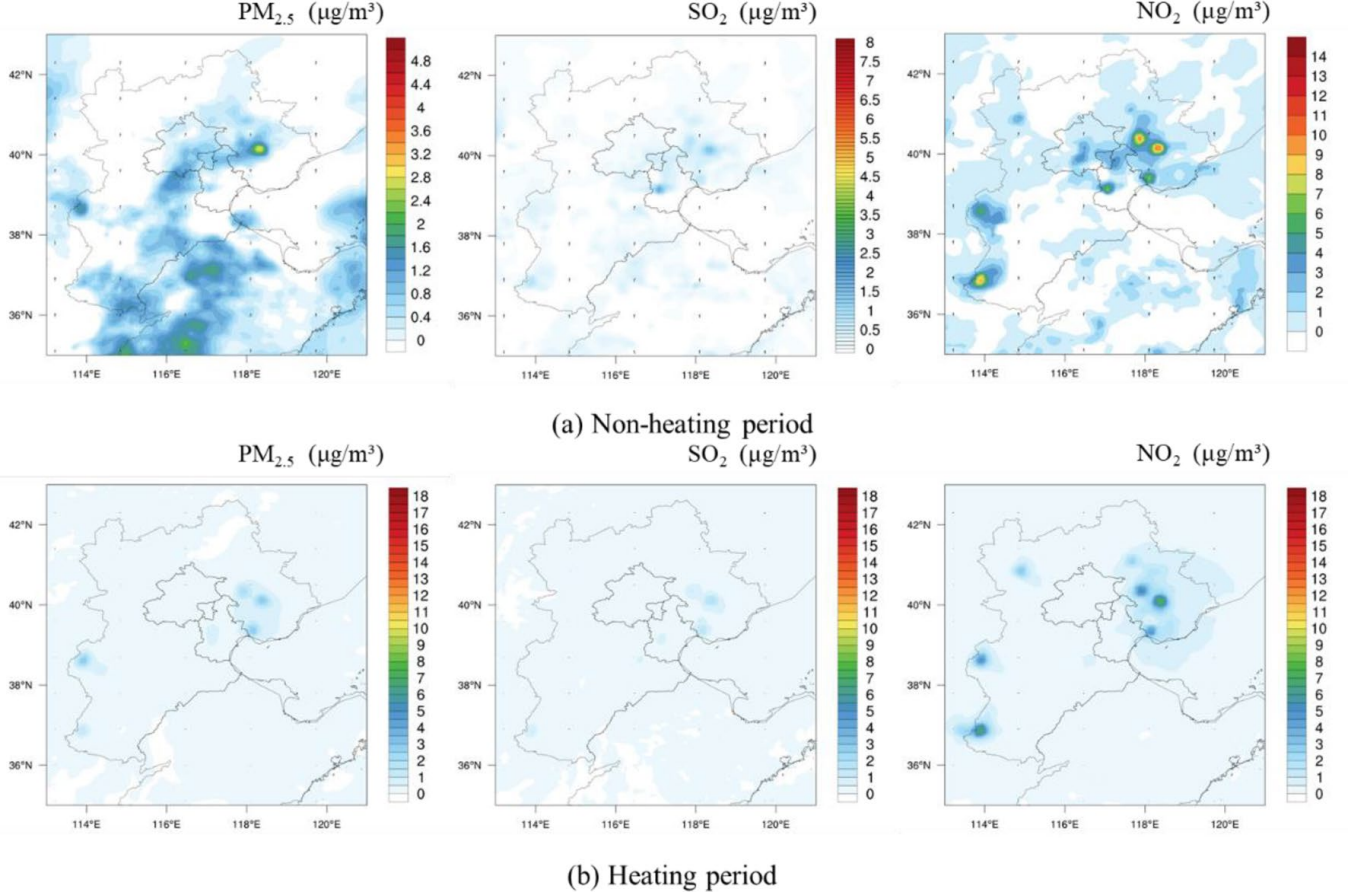
Air quality impacts of iron and steel industry emissions under synergistic emission reduction scenario in BTH region. *Note*: This figure was generated by the authors by using NCAR Command Language (Vision 6.4) (URL: 10.5065/D6WD3XH5).

Figure 12 showed the spatial distribution of the impacts on regional air quality (PM_2.5_, SO_2_, NO_2_) under policy of synergistic reduction of pollution and carbon (Eq. 12). Compared to the baseline scenario, the contribution of the iron and steel industry to air quality significantly decreased under the pollution reduction and carbon reduction scenario. In the non-heating season, the pollution abatement resulted in the reduction of 9.2 μg/m^3^, 11.75 μg/m^3^, and 15.5 μg/m^3^ in the average PM_2.5_, SO_2_, and NO_2_ concentration across the modelled region, respectively, while 20 μg/m^3^ PM_2.5_, 19 μg/m^3^ SO_2_, and 17 μg/m^3^ NO_2_ were reduced during the heating season. Although intensive iron and steel operations in Tangshan, Shijiazhuang and Handan, their contributions of to total iron and steel emission were limited. As Table 6 shown, in the non-heating season, the contribution of the iron and steel industry in Beijing, Shijiazhuang, and Tangshan to the local PM_2.5_ concentration was reduced by 2.08 μg/m^3^, 13.1 μg/m^3^, and 18.65 μg/m^3^, respectively, corresponding to a 51.61%, 67.48%, and 64.55% decrease compared with base scenario. The SO_2_ concentration was reduced by 1.93 μg/m^3^, 20.58 μg/m^3^, 17.62 μg/m^3^ in the three cities, resulting in 59.36%, 85.45%, 58.85% decrease, respectively. For NO_2_, the concentration was reduced by 2.47 μg/m^3^, 36.31 μg/m^3^, and 13.8 μg/m^3^, respectively, corresponding to a decrease by 39.25%, 60.92%, and 37.14%, respectively. During the heating period, the contribution of the iron and steel industry in Beijing, Shijiazhuang, and Tangshan to local PM_2.5_ was reduced by 9.93 μg/m^3^, 10.27 μg/m^3^, and 29.79 μg/m^3^, respectively. The contribution to SO_2_ concentration was reduced by 10.44 μg/m^3^, 15.04 μg/m^3^, and 21 μg/m^3^, respectively. The contribution to NO_2_ concentration decreased by 13.48 μg/m^3^, 31.21 μg/m^3^, and 21.07 μg/m^3^ With the implementation of ultra-low emission retrofits and synergistic control measures, pollutant concentration had reduced more substantially.

## Conclusions

In this study, the emissions of iron and steel industry in the BTH region were calculated by emission factor method. In 2015, the emission of SO_2_, NOx, PM_2.5_, VOC, CO, CO_2_ were 307.3 Kt, 296.0 Kt, 406.2 Kt, 235.4 Kt, 10,229.2 Kt, and 340,459.9 Kt. Among them, sintering, pelletizing, coking and ironmaking were the main emission sources. Tangshan and Handan were the largest emission contributors with the highest air pollutant emission associated with iron and steel industry in the BTH region. After implementation of ultra-low emissions policy, the emission of pollutants in the production chain significantly decreased.

On this basis, the study evaluated the effectiveness of synergistic control and economic cost of 10 typical emission abatement technologies. The technologies were selected from three aspects: optimization of industrial structure, adjustment of energy ratios, and implementation of energy-saving and emission reduction measures. The results showed that the technologies exhibited synergistic emission reduction effects on SO_2_, NOx, PM_2.5_ and CO_2_ emission. Flue gas waste heat recovery, dehumidification blast technology for blast furnace, TRT, CCPP, dry recovery technology for converter gas and process control for hot rolling mills were highlighted with negative marginal cost, indicating greater economic efficiency. Scrap iron-electric arc furnace steelmaking and hydrogen steelmaking were more costly but demonstrated higher potential for emission reduction, which should also be widely promoted.

Moreover, WRF–chem model was used to explore the impact on air quality of iron steel emission under different scenarios. According to the simulation, emission mainly occurred around the cities with a high amount of iron and steel production, such as Tangshan, Shijiazhuang and Handan. Under the baseline scenario, the pollutant emissions from the iron and steel industry for PM_2.5_, SO_2_, and NO_2_ in the BTH region ranges from 0–24 μg/m^3^, 0–28 μg/m^3^, and 0–44 μg/m^3^, respectively, during the non-heating period. During the heating period, the concentration ranged from 0–46 μg/m^3^, 0–42 μg/m^3^, and 0–42 μg/m^3^, respectively. By 2030, after implementation of the ultra-low emission policy and typical synergistic reduction technologies, it would be likely that the contribution of the iron and steel industry to air quality significant decreased. For future efforts, it would be crucial to promote abatement measures with high abatement cost and perform pilot program after achieve cost reductions.

## Data Availability

The emission of pollutants was obtained from the meic inventory (http://meicmodel.org.cn) and the relevant government annual report (https://www.mee.gov.cn). The datasets used and/or analysed during the current study are available from corresponding author on reasonable request.
